# The symmetric ADMM with indefinite proximal regularization and its application

**DOI:** 10.1186/s13660-017-1447-3

**Published:** 2017-07-21

**Authors:** Hongchun Sun, Maoying Tian, Min Sun

**Affiliations:** 1 School of Sciences, Linyi University, Linyi, Shandong 276005 P.R. China; 2 Department of Physiology, Shandong Coal Mining Health School, Taozhuang, Shandong 277011 P.R. China; 3 School of Mathematics and Statistics, Zaozhuang University, Zaozhuang, Shandong 277160 P.R. China; 4 School of Management, Qufu Normal University, Qufu, Shandong 276826 P.R. China

**Keywords:** 90C25, 94A08, symmetric alternating direction method of multipliers, indefinite proximal regularization, global convergence

## Abstract

Due to updating the Lagrangian multiplier twice at each iteration, the symmetric alternating direction method of multipliers (S-ADMM) often performs better than other ADMM-type methods. In practical applications, some proximal terms with positive definite proximal matrices are often added to its subproblems, and it is commonly known that large proximal parameter of the proximal term often results in ‘too-small-step-size’ phenomenon. In this paper, we generalize the proximal matrix from positive definite to indefinite, and propose a new S-ADMM with indefinite proximal regularization (termed IPS-ADMM) for the two-block separable convex programming with linear constraints. Without any additional assumptions, we prove the global convergence of the IPS-ADMM and analyze its worst-case $\mathcal{O}(1/t)$ convergence rate in an ergodic sense by the iteration complexity. Finally, some numerical results are included to illustrate the efficiency of the IPS-ADMM.

## Introduction

Let $\mathcal{R}^{n_{i}}$ stand for an $n_{i}$-dimensional Euclidean space, and let $\mathcal{X}_{i}\subseteq\mathcal{R}^{n_{i}}$ be nonempty, closed and convex set, where $i=1,2$. For two continuous closed convex functions $\theta_{i}(x_{i}): \mathcal{R}^{n_{i}}\rightarrow\mathcal{R}$ ($i=1,2$), the canonical two-block separable convex programming with linear equality constraints is

1 $$ \min \bigl\{ \theta_{1}(x_{1})+ \theta_{2}(x_{2})|A_{1}x_{1}+A_{2}x_{2}=b,{x_{1} \in \mathcal{X}_{1},x_{2}\in\mathcal{X}_{2}} \bigr\} , $$

where $A_{i}\in\mathcal{R}^{m\times n_{i}}$ ($i=1,2$), $b\in\mathcal{R}^{m}$. Throughout, the solution set of (1) is assumed to be nonempty. Convex programming (1) has promising applicability in modeling many concrete problems arising in a wide range of disciplines, such as statistical learning, inverse problems and image processing; see, *e.g.* [1–3] for more details.

Convex programming (1) has been studied extensively in the literature, researchers have developed many numerical methods to solve it during the last decades, which are mainly based on the well-known Douglas-Rachford splitting method [4, 5] and the Peachmen-Rachford splitting method [5, 6], which originate with the partial differential equation (PDE) literature. Concretely, applying the Douglas-Rachford splitting method to the dual of (1) [7, 8], we get the well-known alternating direction of multipliers (ADMM) [9, 10], whose iterative schemes reads

2 $$ \textstyle\begin{cases} x_{1}^{k+1}\in\operatorname{argmin}_{x_{1}\in\mathcal{X}_{1}}\{ \theta_{1}(x_{1})-(\lambda^{k})^{\top}(A_{1}x_{1}+A_{2}x_{2}^{k}-b) \\ \hphantom{x_{1}^{k+1}\in{}}{}+\frac{\beta}{2}\| A_{1}x_{1}+A_{2}x_{2}^{k}-b\|^{2}\}, \\ x_{2}^{k+1}\in\operatorname{argmin}_{x_{2}\in\mathcal{X}_{2}}\{\theta _{2}(x_{2})-(\lambda^{k})^{\top}(A_{1}x_{k}^{k+1}+A_{2}x_{2}-b) \\ \hphantom{x_{2}^{k+1}\in{}}{}+\frac{\beta}{2}\| A_{1}x_{1}^{k+1}+A_{2}x_{2}-b\|^{2}\}, \\ \lambda^{k+1}=\lambda^{k}-s\beta(A_{1}x_{1}^{k+1}+A_{2}x_{2}^{k+1}-b), \end{cases} $$

where $\lambda\in\mathcal{R}^{m}$ is the Lagrangian multiplier; $\beta>0$ is a penalty parameter, and $s\in(0,\frac{1+\sqrt{5}}{2})$ is a relaxation factor. Analogously, applying the Peachmen-Rachford splitting method to the dual of (1), we get the symmetric ADMM [11–13], which generates its sequence via the scheme

3 $$ \textstyle\begin{cases} x_{1}^{k+1}\in\operatorname{argmin}_{x_{1}\in\mathcal{X}_{1}}\{ \theta_{1}(x_{1})-(\lambda^{k})^{\top}(A_{1}x_{1}+A_{2}x_{2}^{k}-b) \\ \hphantom{x_{1}^{k+1}\in{}}{}+\frac{\beta}{2}\| A_{1}x_{1}+A_{2}x_{2}^{k}-b\|^{2}\}, \\ \lambda^{k+\frac{1}{2}}=\lambda^{k}-r\beta(A_{1}x_{1}^{k+1}+A_{2}x_{2}^{k}-b), \\ x_{2}^{k+1}\in\operatorname{argmin}_{x_{2}\in\mathcal{X}_{2}}\{\theta _{2}(x_{2})-(\lambda^{k+\frac{1}{2}})^{\top}(A_{1}x_{k}^{k+1}+A_{2}x_{2}-b) \\ \hphantom{x_{2}^{k+1}\in{}}{}+\frac {\beta}{2}\|A_{1}x_{1}^{k+1}+A_{2}x_{2}-b\|^{2}\}, \\ \lambda^{k+1}=\lambda^{k+\frac{1}{2}}-s\beta(A_{1}x_{1}^{k+1}+A_{2}x_{2}^{k+1}-b), \end{cases} $$

where the feasible region of *r*, *s* is

4 $$ \mathcal{D}=\biggl\{ (r,s)\Big|r\in(-1,1),s\in\biggl(0, \frac {1+\sqrt{5}}{2}\biggr) \ \&\ r+s>0,|r|< 1+s-s^{2}\biggr\} . $$

Both methods make full use of the separable structure of (1), and minimize the primal variables $x_{1}$ and $x_{2}$ individually in the Gauss-Seidel way. As elaborated in [13], the S-ADMM updates the Lagrangian multiplier twice at each iteration and thus the variables $x_{1}$, $x_{2}$ are treated in a symmetric manner. The S-ADMM includes some well-known ADMM-based schemes as special cases. For example, it reduces to the original ADMM (2) when $r=0$, and reduces to the generalized ADMM [14] when $r\in (-1,1)$, $s=1$. Therefore, the S-ADMM provides a unified framework to study the ADMM-type methods. The convergence results of the S-ADMM with any $(r,s)\in\mathcal{D}$, including global convergence, the worst-case $\mathcal{O}(1/t)$ convergence rate in an ergodic sense, have been established in [13]. To the best of the authors’ knowledge, the worst-case $\mathcal{O}(1/t)$ convergence rate in some non-ergodic sense of the S-ADMM is still missing.

In practical applications, the two essential subproblems related to $x_{1}$ and $x_{2}$ dominate the computation of the S-ADMM, which are often either linear or easily solvable, but nevertheless challenging. In order to solve the issue, some proximal terms are often added to these subproblems, which can linearize the quadratic term $\frac{\beta }{2}\|A_{i}x_{i}\|^{2}$ ($i=1,2$) of these subproblems, and as a result we have the following proximal S-ADMM (termed PS-ADMM) [15–17]:

5 $$ \textstyle\begin{cases}x_{1}^{k+1}\in\operatorname{argmin}_{x_{1}\in\mathcal{X}_{1}}\{ \theta_{1}(x_{1})-(\lambda^{k})^{\top}(A_{1}x_{1}+A_{2}x_{2}^{k}-b) \\ \hphantom{x_{1}^{k+1}\in{}}{}+\frac{\beta}{2}\| A_{1}x_{1}+A_{2}x_{2}^{k}-b\|^{2}\}, \\ \lambda^{k+\frac{1}{2}}=\lambda^{k}-r\beta(A_{1}x_{1}^{k+1}+A_{2}x_{2}^{k}-b), \\ x_{2}^{k+1}\in\operatorname{argmin}_{x_{2}\in\mathcal{X}_{2}}\{\theta _{2}(x_{2})-(\lambda^{k+\frac{1}{2}})^{\top}(A_{1}x_{k}^{k+1}+A_{2}x_{2}-b) \\ \hphantom{x_{2}^{k+1}\in{}}{}+\frac{\beta}{2}\|A_{1}x_{1}^{k+1}+A_{2}x_{2}-b\|^{2}+\frac{1}{2}\|x_{2}-x_{2}^{k}\| _{G}^{2}\}, \\ \lambda^{k+1}=\lambda^{k+\frac{1}{2}}-s\beta(A_{1}x_{1}^{k+1}+A_{2}x_{2}^{k+1}-b), \end{cases} $$

where $G\in\mathcal{R}^{n_{2}\times n_{2}}$ is a positive definite matrix. When we set $G=\tau I_{n_{2}}-\beta A_{2}^{\top}A_{2}$ with $\tau>\beta\| A_{2}^{\top}A_{2}\|$, the quadratic term $\frac{\beta}{2}\|A_{2}x_{2}\|^{2}$ in the subproblem related to $x_{2}$ of the PS-ADMM is offset and thus the quadratic term $\frac{\beta}{2}\|A_{1}x_{1}^{k+1}+A_{2}x_{2}-b\|^{2}$ is linearized. Then, if $\mathcal{X}_{2}=\mathcal{R}^{n_{2}}$, the PS-ADMM only needs to compute the proximal mapping of the involved convex function $\theta_{2}(\cdot)$ at each iteration, which is often simple enough to have a closed-form solution in many practical applications, such as $\theta_{2}(x_{2})=\|x_{2}\|_{1}$ in the compressive sensing problems [3], $\theta_{2}(x_{2})=\|x_{2}\|_{*}$ (here $x_{2}$ is a square matrix) in the robust principal component analysis models [18]. $\|x_{2}\|_{*}$ is defined by the sum of all singular values of $x_{2}$.

The curse accompanying the above improvement in solvability is that the proximal parameter *τ* is not easy to determine for some problems in practice. Large *τ* prompts the weight of the quadratic term $\frac{1}{2}\|x_{2}-x_{2}^{k}\|_{G}^{2}$ in the objective function of the $x_{2}$-subproblem and inevitably results in the ‘too-small-step-size’ phenomenon. Then, the advance of $x_{2}$ is tiny at the *k*th iteration, which often slows down the convergence of the corresponding method. Therefore, it is meaningful to expand the feasible set of *τ*. Obviously, if we further reduce *τ* to $\tau\leq\beta\|A_{2}^{\top}A_{2}\| $, the proximal matrix *G* will become indefinite, and it is thus natural to ask whether or not the corresponding method with such *G* is still globally convergent? Quite recently the authors in [19–21] partially answered the question. More specifically, for the ADMM (2) with ${s}=1$, He *et al.* [19] have proved that the feasible set of *τ* can be expanded to $\{\tau |\tau>0.8\beta\|A_{2}^{\top}A_{2}\|\}$, and for the ADMM (2) with ${s}\in(0,\frac{1+\sqrt{5}}{2})$, Sun *et al.* [20] have proved that the feasible set of *τ* can be expanded to $\{\tau|\tau>(5-\min\{{s,1+s-s^{2}}\})\beta\| A_{2}^{\top}A_{2}\|/5\}$. Then, for the S-ADMM with $r\in(-1,1)$, $s=1$, Gao *et al.* [21] have proved that the feasible set of *τ* can be expanded to $\{\tau|\tau>(r^{2}-r+4)\beta\|A_{2}^{\top}A_{2}\|/(r^{2}-2r+5)\}$. Other relevant studies can be found in [22, 23]. In this paper, we continue to study along this direction, and present a new feasible set of *τ*, which generalizes those in [19–21] to any $(r,s)\in\mathcal{D}$. Furthermore, we show that for any $(r,s)\in\mathcal{D}$, the global convergence of the S-ADMM with some indefinite proximal regularization can be guaranteed.

The rest of the paper is organized as follows. In Section 2, we summarize some preliminaries which are useful for further discussion. Then, in Section 3, we list the iterative scheme of the IPS-ADMM and prove its convergence results, including the global convergence and the convergence rate. Some preliminary numerical results are reported in Section 4. Finally, some conclusions are drawn in Section 5.

## Preliminaries

In this section, we first list some notation used in this paper, and then characterize problem (1) by a mixed variational inequality problem. Some matrices and variables to simplify the notation of our later analysis are also defined.

For any two vectors $x,y\in\mathcal{R}^{n}$, $\langle x,y\rangle$ or $x^{\top}y$ denote their inner product. For any two matrices $A\in \mathcal{R}^{s\times m}$, $B\in\mathcal{R}^{n\times s}$, the Kronecker product of *A* and *B* is defined as $A\otimes B=(a_{ij}B)$. We let $\|\cdot\|_{1}$ and $\|\cdot\|$ be the $\ell_{1}$-norm and $\ell_{2}$-norm for vector variables, respectively. $I_{n}$ denotes the *n*-dimensional identity matrix. If the matrix $G\in \mathcal{R}^{n\times n}$ is symmetric, we use the symbol $\|x\|_{G}^{2}$ to denote $x^{\top}Gx$ even if *G* is indefinite; $G\succ0$ (resp., $G\succeq0$) denotes that the matrix *G* is positive definite (resp., semi-definite).

Let us split the feasible set $\mathcal{D}$ of the parameters $(r,s)$ into the following five subsets:

6 $$ \textstyle\begin{cases} \mathcal{D}_{1}=\{(r,s)|r\in(-1,1),s\in(0,1),r+s>0\}, \\ \mathcal{D}_{2}=\{(r,s)|r\in(-1,1),s=1\}, \\ \mathcal{D}_{3}=\{(r,s)|r=0,s\in(1,\frac{1+\sqrt{5}}{2})\}, \\ \mathcal{D}_{4}=\{(r,s)|r\in(0,1),s\in(1,\frac{1+\sqrt{5}}{2}) \ \&\ r< 1+s-s^{2}\}, \\ \mathcal{D}_{5}=\{(r,s)|r\in(-1,0),s\in(1,\frac{1+\sqrt{5}}{2}) \ \&\ -r< 1+s-s^{2}\}. \end{cases} $$

Obviously, the set $\{\mathcal{D}_{1},\mathcal{D}_{2},\mathcal{D}_{3},\mathcal {D}_{4},\mathcal{D}_{5}\}$ is a simplicial partition of the set $\mathcal{D}$.

Throughout, the proximal matrix *G* is defined by

7 $$ G=\tau I_{n_{2}}-\beta A_{2}^{\top}A_{2}, $$

where we set $\tau=\alpha\tilde{\tau}$ with $\tilde{\tau}>\beta\| A_{2}^{\top}A_{2}\|$, $\alpha\in(c(r,s),+\infty)$, and $c(r,s)$ is defined by

8 $$ c(r,s)= \textstyle\begin{cases} s+\frac{(1-s)^{2}}{2-r-s},& \mbox{if } (r,s)\in\mathcal{D}_{1}, \\ \frac{4-r-r^{2}}{5-3r},& \mbox{if } (r,s)\in\mathcal{D}_{2}, \\ \frac{7s^{2}-22s+23}{5s^{2}-20s+25},& \mbox{if } (r,s)\in\mathcal{D}_{3}, \\ \frac{r^{3} + r^{2} - r - 5}{3r^{2} - 2r - 5},& \mbox{if } (r,s)\in\mathcal {D}_{4}, \\ \frac{(r^{2} + r - 4)s^{2} -( r^{2} +4r - 9)s - (r-1)^{2}}{s(2-s)(5-3r)}, &\mbox{if } (r,s)\in\mathcal{D}_{5}. \end{cases} $$

### Remark 2.1

Note that $c(r,s)\leq1$ if $(r,s)\in\mathcal{D}$; see Lemmas 3.4-3.8 in Section 3. Therefore, the feasible set of *τ* is expanded from $\{\tau|\tau>\beta\|A_{2}^{\top}A_{2}\|\}$ to $\{\tau|\tau>c(r,s)\beta\| A_{2}^{\top}A_{2}\|\}$, which provides more choices for researchers or practitioners.

Furthermore, we define an auxiliary matrix as follows:

9 $$ G_{0}=\alpha\bigl(\tilde{\tau} I_{n_{2}}-\beta A_{2}^{\top}A_{2}\bigr), $$

which is positive definite by $\tilde{\tau}>\beta\|A_{2}^{\top}A_{2}\|$.

Invoking the first-order optimality condition for convex programming, we get the following equivalent form of problem (1): Finding a vector $w^{*}\in\mathcal{W}$ such that

10 $$ \theta(x)-\theta\bigl(x^{*}\bigr)+\bigl(w-w^{*}\bigr)^{\top}F \bigl(w^{*}\bigr)\geq0,\quad \forall w\in\mathcal{W}, $$

where

11 $$\begin{aligned}& x=\left ( \textstyle\begin{array}{@{}c@{}}x_{1}\\ x_{2} \end{array}\displaystyle \right ),\qquad w=\left ( \textstyle\begin{array}{@{}c@{}}x_{1}\\ x_{2}\\ \lambda \end{array}\displaystyle \right ),\qquad \theta(x)= \theta_{1}(x_{1})+\theta_{2}(x_{2}), \\& F(w)=\left ( \textstyle\begin{array}{@{}c@{}}-A_{1}^{\top}\lambda\\ -A_{2}^{\top}\lambda\\ A_{1}x_{1}+A_{2}x_{2}-b \end{array}\displaystyle \right ),\qquad \mathcal{W}= \mathcal{X}_{1}\times\mathcal{X}_{2}\times \mathcal{R}^{m}. \end{aligned}$$

Obviously, the problem (10) is a mixed variational inequality problem, which is denoted by $\operatorname{MVI}(\theta, F,\mathcal{W})$. The mapping $F(w)$ defined in (11) is not only monotone, but also satisfies the property

12 $$ w^{\top}\bigl(F(w)-F(\tilde{w})\bigr)= \tilde{w}^{\top}\bigl(F(w)-F(\tilde{w})\bigr),\quad \forall w,\tilde{w} \in\mathcal{R}^{m+n_{1}+n_{2}}. $$

Furthermore, the solution set of $\operatorname{MVI}(\theta, F,\mathcal{W})$, denoted by $\mathcal{W}^{*}$, is nonempty under the nonempty assumption for the solution set of problem (1).

Now, let us define three matrices in order to make our following analysis more succinct. Set

13 $$\begin{aligned}& M=\left ( \textstyle\begin{array}{@{}c@{\quad}c@{\quad}c@{}} I_{n_{1}}&0&0\\ 0&I_{n_{2}}&0\\ 0&-s\beta A_{2}&(r+s)I_{m} \end{array}\displaystyle \right ),\qquad Q= \left ( \textstyle\begin{array}{@{}c@{\quad}c@{\quad}c@{}} P&0&0\\ 0&G+\beta A_{2}^{\top}A_{2}&-rA_{2}^{\top}\\ 0&-A_{2}&\frac{1}{\beta}I_{m} \end{array}\displaystyle \right ), \end{aligned}$$

14 $$\begin{aligned}& H=\left ( \textstyle\begin{array}{@{}c@{\quad}c@{\quad}c@{}} P&0&0\\ 0&G+(1-\frac{rs}{r+s})\beta A_{2}^{\top}A_{2}&-\frac{r}{r+s}A_{2}^{\top}\\ 0&-\frac{r}{r+s}A_{2}&\frac{1}{(r+s)\beta}I_{m} \end{array}\displaystyle \right ). \end{aligned}$$

### Lemma 2.1

*Suppose the matrix* $A_{2}$ *is full column rank and the parameter* *α* *in* (7) *satisfies*

15 $$ \alpha>\alpha_{0}\doteq\frac{rs+r^{2}}{r+s}. $$

*Then*, *the matrices* *M*, *Q*, *H* *defined*, *respectively*, *in* (6), (7) *satisfies*

16 $$\begin{aligned}& HM=Q, \end{aligned}$$

17 $$\begin{aligned}& H\succ0. \end{aligned}$$

### Proof

The proof of (16) is trivial, and we only need to prove (17). By the positive definiteness of *P*, we only need to prove $H(2:3,2:3)$ is positive definite. Here $H(2:3,2:3)$ denotes the corresponding sub-matrix formed from the rows and columns with the indices $(2:3)$ and $(2:3)$ as in Matlab. Substituting (7) into the right-hand side of (14), we get

$$\begin{aligned} H(2:3,2:3) =&\left ( \textstyle\begin{array}{@{}c@{\quad}c@{}} \alpha\tilde{\tau} I_{n_{2}}-\frac{rs}{r+s}\beta A_{2}^{\top}A_{2}&-\frac {r}{r+s}A_{2}^{\top}\\ -\frac{r}{r+s}A_{2}&\frac{1}{(r+s)\beta}I_{m} \end{array}\displaystyle \right ) \\ =&\left ( \textstyle\begin{array}{@{}c@{\quad}c@{}} \alpha(\tilde{\tau} I_{n_{2}}-\beta A_{2}^{\top}A_{2})&0\\ 0&0 \end{array}\displaystyle \right )+\left ( \textstyle\begin{array}{@{}c@{\quad}c@{}} (\alpha-\frac{rs}{r+s})\beta A_{2}^{\top}A_{2}&-\frac{r}{r+s}A_{2}^{\top}\\ -\frac{r}{r+s}A_{2}&\frac{1}{(r+s)\beta}I_{m} \end{array}\displaystyle \right ) \\ \succeq&\left ( \textstyle\begin{array}{@{}c@{\quad}c@{}} (\alpha-\frac{rs}{r+s})\beta A_{2}^{\top}A_{2}&-\frac{r}{r+s}A_{2}^{\top}\\ -\frac{r}{r+s}A_{2}&\frac{1}{(r+s)\beta}I_{m} \end{array}\displaystyle \right ) \\ =&\frac{1}{r+s}\left ( \textstyle\begin{array}{@{}c@{\quad}c@{}} A_{2}^{\top}&0\\ 0&I_{m} \end{array}\displaystyle \right )\left ( \textstyle\begin{array}{@{}c@{\quad}c@{}} \beta((r+s)\alpha-rs)I_{m}&-rI_{m}\\ -rI_{m}&\frac{1}{\beta}I_{m} \end{array}\displaystyle \right )\left ( \textstyle\begin{array}{@{}c@{\quad}c@{}} A_{2}&0\\ 0&I_{m} \end{array}\displaystyle \right ), \end{aligned}$$

where the relationship ⪰ comes from $\alpha>0$ and $\tilde{\tau }>\beta\|A_{2}^{\top}A_{2}\|$. Since the matrix $A_{2}$ is full column rank, we only need to prove the positive definiteness of the matrix

$$\left ( \textstyle\begin{array}{@{}c@{\quad}c@{}} \beta((r+s)\alpha-rs)I_{m}&-rI_{m}\\ -rI_{m}&\frac{1}{\beta}I_{m} \end{array}\displaystyle \right ), $$

which can be further written as

$$\left ( \textstyle\begin{array}{@{}c@{\quad}c@{}} \beta((r+s)\alpha-rs)&-r\\ -r&\frac{1}{\beta} \end{array}\displaystyle \right )\otimes I_{m}, $$

where ⊗ denotes the matrix Kronecker product. Then, we only need to show the 2-by-2 matrix

$$\left ( \textstyle\begin{array}{@{}c@{\quad}c@{}} \beta((r+s)\alpha-rs)&-r\\ -r&\frac{1}{\beta} \end{array}\displaystyle \right ) $$

is positive definite. In fact, by (15), we have

$$\beta\bigl((r+s)\alpha-rs\bigr)\times\frac{1}{\beta}-r^{2}=(r+s) \alpha-rs-r^{2}>0. $$

Therefore, the matrix *H* is positive definite. The proof is completed. □

At the end of this section, let us summarize two criteria to measure the worst-case $\mathcal{O}(1/t)$ convergence rate of the ADMM-type methods in an ergodic sense.

1. For a given compact set $\bar{\mathcal{D}}\subset\mathcal {R}^{m+n}$, let $d=\sup\{\|w-w^{0}\||w\in\bar{\mathcal{D}}\}$, where $w^{0}$ is the initial iterate. He *et al.* [24] established the following criterion:
  18 $$ \sup_{w\in\bar{\mathcal{D}}}\bigl\{ \theta (x_{t})- \theta(x)+(w_{t}-w)^{\top}F(w)\bigr\} \leq\frac{Cd^{2}}{t+1}, $$
  where $w_{t}=\frac{1}{t+1}\sum_{k=0}^{t}w^{k}$, $C>0$, and *t* is the iteration counter. This criterion is used in [19, 21]. Obviously, we can only ensure that any $w\in\bar{\mathcal{D}}$ satisfies (18). Therefore, the criterion (18) is not reasonable.
2. In [25], Lin *et al.* proposed the following criterion:
  19 $$ \theta(x_{t})-\theta\bigl(x^{*}\bigr)+ \bigl(x_{t}-x^{*}\bigr)^{\top}\bigl(-A^{\top}\lambda^{*} \bigr)+\frac{c}{2}\|Ax_{t}-b\|^{2}\leq \frac{C}{t+1}, $$
  where $c>0$. Proposition 1 in [25] indicates that the vector ${x}_{t}\in\mathcal{X}_{1}\times\mathcal{X}_{2}$ is an optimal solution to (1) if and only if the left-hand side of (19) equals zero. Compared with (18), the criterion (19) is more reasonable. Therefore, we shall use a criterion similar to (19) to measure the $\mathcal{O}(1/t)$ convergence rate of our new method.

## Algorithm and convergence results

In this section, we first present the symmetric ADMM with indefinite proximal regularization (termed IPS-ADMM), and then prove the convergence results of the sequence generated by the IPS-ADMM.

### Algorithm 3.1

The IPS-ADMM for problem (1)

Step 0.: Input four parameters $(r,s)\in\mathcal{D}$, $\alpha\in (c(r,s),+\infty)$, $\beta>0$, the tolerance $\varepsilon>0$, and the proximal matrices $P\in\mathcal{R}^{n_{1}\times n_{1}}$ with $P\succ0$ and $G\in\mathcal{R}^{n_{2}\times n_{2}}$ defined by (7). Initialize $(x_{1},x_{2},\lambda):=(x_{1}^{0},x_{2}^{0},\lambda^{0})$, and set $k:=0$.
Step 1.: Compute the new iterate $w^{k+1}=(x_{1}^{k+1},x_{2}^{k+1},\lambda^{k+1})$ by the following iterative scheme:
  20 $$ \textstyle\begin{cases} x_{1}^{k+1}\in\operatorname{argmin}_{x_{1}\in\mathcal{X}_{1}}\{ \theta_{1}(x_{1})-(\lambda^{k})^{\top}(A_{1}x_{1}+A_{2}x_{2}^{k}-b) \\ \hphantom{x_{1}^{k+1}\in{}}{}+\frac{\beta}{2}\| A_{1}x_{1}+A_{2}x_{2}^{k}-b\|^{2}+\frac{1}{2}\|x_{1}-x_{1}^{k}\|_{P}^{2}\}, \\ \lambda^{k+\frac{1}{2}}=\lambda^{k}-r\beta(A_{1}x_{1}^{k+1}+A_{2}x_{2}^{k}-b), \\ x_{2}^{k+1}\in\operatorname{argmin}_{x_{2}\in\mathcal{X}_{2}}\{\theta _{2}(x_{2})-(\lambda^{k+\frac{1}{2}})^{\top}(A_{1}x_{k}^{k+1}+A_{2}x_{2}-b) \\ \hphantom{x_{2}^{k+1}\in{}}{}+\frac{\beta}{2}\|A_{1}x_{1}^{k+1}+A_{2}x_{2}-b\|^{2}+\frac{1}{2}\|x_{2}-x_{2}^{k}\| _{G}^{2}\}, \\ \lambda^{k+1}=\lambda^{k+\frac{1}{2}}-s\beta(A_{1}x_{1}^{k+1}+A_{2}x_{2}^{k+1}-b). \end{cases} $$
Step 2.: If $\|w^{k}-{w}^{k+1}\|\leq\varepsilon$, then stop; otherwise set $k:=k+1$, and go to Step 1.

### Remark 3.1

Since the global convergence of IPS-ADMM with $\alpha\geq1$ has been established in the literature [16, 26–28], in the following, we restrict $\alpha\in (c(r,s),1)$.

To prove the convergence results of the IPS-ADMM, we first define a block matrix and an auxiliary variable.

$$A=(A_{1},A_{2}), \qquad \tilde{w}^{k}=\left ( \textstyle\begin{array}{@{}c@{}}\tilde{x}_{1}^{k}\\ \tilde{x}_{2}^{k}\\ \tilde{\lambda}^{k} \end{array}\displaystyle \right )=\left ( \textstyle\begin{array}{@{}c@{}}x_{1}^{k+1}\\ x_{2}^{k+1}\\ \lambda^{k}-\beta(A_{1}x_{1}^{k+1}+A_{2}x_{2}^{k}-b) \end{array}\displaystyle \right ). $$

### Lemma 3.1

*For the sequence* $\{(x^{k},\lambda^{k})\}=\{ (x_{1}^{k},x_{2}^{k},\lambda^{k})\}$ *generated by the IPS*-*ADMM*, *we have*

21 $$ \theta(x)-\theta\bigl(x^{k+1}\bigr)+\bigl(w-\tilde {w}^{k}\bigr)^{\top}F\bigl(\tilde{w}^{k}\bigr)\geq \bigl(w-\tilde{w}^{k}\bigr)^{\top}Q\bigl(w^{k}- \tilde{w}^{k}\bigr),\quad \forall w\in\mathcal{W}, $$

*and*

22 $$\begin{aligned}& \theta(x)-\theta\bigl(x^{k+1}\bigr)+\bigl(w-\tilde {w}^{k}\bigr)^{\top}F(w) \\& \quad \geq\frac{1}{2}\bigl( \bigl\Vert w-w^{k+1} \bigr\Vert ^{2}_{H}- \bigl\Vert w-w^{k} \bigr\Vert _{H}^{2}\bigr)+ \frac {1}{2} \bigl\Vert w^{k}-\tilde{w}^{k} \bigr\Vert _{R}^{2},\quad \forall w\in\mathcal{W}, \end{aligned}$$

*where* $R=Q^{\top}+Q-M^{\top}HM$.

### Proof

The proof of this lemma is similar to that of Lemma 3.1 and Theorem 4.2 in [13], which is omitted. □

### Remark 3.2

By the definition of $F(\cdot)$ in (11), (12), for any $(x_{1},x_{2},\lambda)\in\mathcal{R}^{m+n_{1}+n_{2}}$ such that $A_{1}x_{1}+A_{2}x_{2}=b$, the left-hand side of (22) can be written as

23 $$\begin{aligned}& \bigl(w-\tilde{w}^{k}\bigr)^{\top}F(w) \\& \quad =\bigl(w-\tilde{w}^{k}\bigr)^{\top}F\bigl( \tilde{w}^{k}\bigr) \\& \quad =\bigl(x_{1}-\tilde{x}_{1}^{k} \bigr)^{\top}\bigl(-A_{1}\tilde{\lambda}^{k}\bigr)+ \bigl(x_{2}-\tilde {x}_{2}^{k}\bigr)^{\top}\bigl(-A_{2}\tilde{\lambda}^{k}\bigr)+\bigl(\lambda-\tilde{ \lambda}^{k}\bigr)^{\top}\bigl(A_{1} \tilde{x}_{1}^{k}+A_{2}\tilde{x}_{2}^{k}-b \bigr) \\& \quad =\bigl(A_{1}\tilde{x}_{1}^{k}+A_{2} \tilde{x}_{2}^{k}-b\bigr)^{\top}\tilde{ \lambda}^{k}+\bigl(\lambda -\tilde{\lambda}^{k} \bigr)^{\top}\bigl(A_{1}\tilde{x}_{1}^{k}+A_{2} \tilde{x}_{2}^{k}-b\bigr) \\& \quad =\lambda^{\top}\bigl(A_{1}\tilde{x}_{1}^{k}+A_{2} \tilde{x}_{2}^{k}-b\bigr) \\& \quad =\lambda^{\top}\bigl(A{x}^{k+1}-Ax\bigr) \\& \quad =\bigl(x-{x}^{k+1}\bigr)^{\top}\bigl(-A^{\top}\lambda\bigr). \end{aligned}$$

Then, substituting the above equality into the left-hand side of (22), we get

24 $$ \theta\bigl(x^{k+1}\bigr)-\theta(x)+\bigl({x}^{k+1}-x \bigr)^{\top}\bigl(-A^{\top}\lambda\bigr)\leq\frac{1}{2} \bigl( \bigl\Vert w-w^{k} \bigr\Vert _{H}^{2}- \bigl\Vert w-w^{k+1} \bigr\Vert ^{2}_{H}\bigr)- \frac {1}{2} \bigl\Vert w^{k}-\tilde{w}^{k} \bigr\Vert _{R}^{2}, $$

where the vector $(x_{1},x_{2},\lambda)\in\mathcal{R}^{m+n_{1}+n_{2}}$ satisfies $A_{1}x_{1}+A_{2}x_{2}=b$.

Comparing all the terms appeared in (19) and (24), we find that the left-hand side of (24) does not have the term $\| Ax^{k+1}-b\|^{2}$ temporarily, and due to the indefinite of *R*, the term $\|v^{k}-\tilde{v}^{k}\|_{R}^{2}$ on the right-hand side of (24) maybe negative. Now let us deal with the term $\|v^{k}-\tilde{v}^{k}\|_{R}^{2}$, and by doing so, the term $\|Ax^{k+1}-b\|^{2}$ will also appear. By a manipulation, we get the concrete expression of the matrix *R*, which is as follows:

25 $$ R=\left ( \textstyle\begin{array}{@{}c@{\quad}c@{\quad}c@{}} P&0&0 \\ 0&G+(1-s)\beta A_{2}^{\top}A_{2}&-(1-s)A_{2}^{\top}\\ 0&-(1-s)A_{2}&\frac{2-(r+s)}{\beta}I_{m} \end{array}\displaystyle \right ). $$

### Lemma 3.2

*Let* $\{(x^{k},\lambda^{k})\}=\{(x_{1}^{k},x_{2}^{k},\lambda^{k})\} $ *be the sequence generated by the IPS*-*ADMM*. *Then we have*

26 $$\begin{aligned}& \bigl\Vert w^{k}-\tilde{w}^{k} \bigr\Vert _{R}^{2} \\& \quad = \bigl\Vert x_{1}^{k}-x_{1}^{k+1} \bigr\Vert _{P}^{2}+ \bigl\Vert x_{2}^{k}-x_{2}^{k+1} \bigr\Vert _{G}^{2}+(1-r)\beta \bigl\Vert A_{2}\bigl(x_{2}^{k}-x_{2}^{k+1} \bigr) \bigr\Vert ^{2} \\& \qquad {} +(2-r-s)\beta \bigl\Vert Ax^{k+1}-b \bigr\Vert ^{2}+2(1-r)\beta\bigl(Ax^{k+1}-b\bigr)^{\top}A_{2}\bigl(x_{2}^{k+1}-x_{2}^{k} \bigr). \end{aligned}$$

### Proof

The proof of this lemma is similar to that of Lemma 5.1 in [13], which is omitted. □

The following lemma deals with the crossing term $(Ax^{k+1}-b)^{\top}A_{2}(x_{2}^{k+1}-x_{2}^{k})$ on the right-hand side of (26), whose proof is mainly motivated by those of Lemma 3.2 in [26] and Lemma 5.2 in [13].

### Lemma 3.3

*Let* $\{(x^{k},\lambda^{k})\}=\{(x_{1}^{k},x_{2}^{k},\lambda^{k})\} $ *be the sequence generated by the IPS*-*ADMM*. *Then we have*

27 $$\begin{aligned}& \bigl(Ax^{k+1}-b\bigr)^{\top}A_{2} \bigl(x_{2}^{k+1}-x_{2}^{k}\bigr) \\& \quad \geq\frac{1-s}{1+r}\bigl(Ax^{k}-b\bigr)^{\top}A_{2}\bigl(x_{2}^{k}-x_{2}^{k+1} \bigr)-\frac{r}{1+r} \bigl\Vert A_{2}\bigl(x_{2}^{k}-x_{2}^{k+1} \bigr) \bigr\Vert ^{2} \\& \qquad {} +\frac{\alpha}{2(1+r)\beta}\bigl( \bigl\Vert x_{2}^{k}-x_{2}^{k+1} \bigr\Vert _{G_{0}}^{2}- \bigl\Vert x_{2}^{k-1}-x_{2}^{k} \bigr\Vert _{G_{0}}^{2}\bigr) \\& \qquad {}-\frac{1-\alpha}{2(1+r)}\bigl(3 \bigl\Vert A_{2}\bigl(x_{2}^{k}-x_{2}^{k+1} \bigr) \bigr\Vert ^{2}+ \bigl\Vert A_{2} \bigl(x_{2}^{k-1}-x_{2}^{k}\bigr) \bigr\Vert ^{2}\bigr). \end{aligned}$$

### Proof

The first-order optimality condition of $x_{2}$-subproblem in (5) indicates that, for any $x_{2}\in\mathcal{X}_{2}$,

28 $$ \theta_{2}(x_{2})-\theta _{2} \bigl({x}_{2}^{k+1}\bigr)+\bigl(x_{2}-{x}_{2}^{k+1} \bigr)^{\top}\bigl\{ -A_{2}^{\top}\lambda^{k+\frac{1}{2}}+ \beta \bigl(A{x}^{k+1}-b\bigr)+G\bigl(x_{2}^{k+1}-x_{2}^{k} \bigr) \bigr\} \geq0. $$

Setting $x_{2}=x_{2}^{k}$ in (28), we get

$$\theta_{2}\bigl(x_{2}^{k}\bigr)- \theta_{2}\bigl({x}_{2}^{k+1}\bigr)+ \bigl(x_{2}^{k}-{x}_{2}^{k+1} \bigr)^{\top}\bigl\{ -A_{2}^{\top}\lambda^{k+\frac{1}{2}}+ \beta \bigl(A{x}^{k+1}-b\bigr)+G\bigl(x_{2}^{k+1}-x_{2}^{k} \bigr) \bigr\} \geq0. $$

Similarly, taking $x_{2}=x_{2}^{k+1}$ in (28) for $k:=k-1$, we have

$$\theta_{2}\bigl({x}_{2}^{k+1}\bigr)- \theta_{2}\bigl(x_{2}^{k}\bigr)+ \bigl({x}_{2}^{k+1}-x_{2}^{k} \bigr)^{\top}\bigl\{ -A_{2}^{\top}\lambda^{k-\frac{1}{2}}+ \beta \bigl(A{x}^{k}-b\bigr)+G\bigl(x_{2}^{k}-x_{2}^{k-1} \bigr) \bigr\} \geq0. $$

Then, adding the above two inequalities, we get

29 $$\begin{aligned}& \bigl(x_{2}^{k}-x_{2}^{k+1} \bigr)^{\top}A_{2}^{\top}\bigl\{ \bigl( \lambda^{k-\frac{1}{2}}-\lambda ^{k+\frac{1}{2}}\bigr)-\beta\bigl(Ax^{k}-b \bigr)+\beta\bigl(Ax^{k+1}-b\bigr)\bigr\} \\& \quad \geq \bigl\Vert x_{2}^{k+1}-x_{2}^{k} \bigr\Vert _{G}^{2}+\bigl(x_{2}^{k}-x_{2}^{k+1} \bigr)^{\top}G\bigl(x_{2}^{k}-x_{2}^{k-1} \bigr). \end{aligned}$$

From the update formula for *λ* in (5), we have

$$\begin{aligned} \lambda^{k+\frac{1}{2}} =&\lambda^{k}-r\beta\bigl(A_{1}x_{1}^{k+1}+A_{2}x_{2}^{k}-b \bigr) \\ =&\lambda^{k-\frac{1}{2}}-s\beta\bigl(A_{1}x_{1}^{k}+A_{2}x_{2}^{k}-b \bigr)-r\beta \bigl(A_{1}x_{1}^{k+1}+A_{2}x_{2}^{k}-b \bigr). \end{aligned}$$

Substituting the above equality into the left-hand side of (29), we get

30 $$\begin{aligned}& \bigl(x_{2}^{k}-x_{2}^{k+1} \bigr)^{\top}A_{2}^{\top}\bigl\{ (1+r)\beta \bigl(Ax^{k+1}-b\bigr)-(1-s)\beta \bigl(Ax^{k}-b\bigr)+r\beta A_{2}\bigl(x_{2}^{k}-x_{2}^{k+1} \bigr)\bigr\} \\& \quad \geq \bigl\Vert x_{2}^{k+1}-x_{2}^{k} \bigr\Vert _{G}^{2}+\bigl(x_{2}^{k}-x_{2}^{k+1} \bigr)^{\top}G\bigl(x_{2}^{k}-x_{2}^{k-1} \bigr). \end{aligned}$$

By the definitions of *G* and $G_{0}$ (see (7) and (9)), we have

$$\begin{aligned}& \bigl\Vert x_{2}^{k+1}-x_{2}^{k} \bigr\Vert _{G}^{2}+\bigl(x_{2}^{k}-x_{2}^{k+1} \bigr)^{\top}G\bigl(x_{2}^{k}-x_{2}^{k-1} \bigr) \\& \quad = \alpha \bigl\Vert x_{2}^{k+1}-x_{2}^{k} \bigr\Vert _{G_{0}}^{2}-(1-\alpha)\beta \bigl\Vert A_{2}\bigl(x_{2}^{k+1}-x_{2}^{k} \bigr) \bigr\Vert ^{2}+\alpha\bigl(x_{2}^{k}-x_{2}^{k+1} \bigr)^{\top}G_{0}\bigl(x_{2}^{k}-x_{2}^{k-1} \bigr) \\& \qquad {}-(1-\alpha)\beta\bigl(A_{2}x_{2}^{k}-A_{2}x_{2}^{k+1} \bigr)^{\top}\bigl(A_{2}x_{2}^{k}-A_{2}x_{2}^{k-1} \bigr) \\& \quad \geq \frac{\alpha}{2}\bigl( \bigl\Vert x_{2}^{k}-x_{2}^{k+1} \bigr\Vert _{G_{0}}^{2}- \bigl\Vert x_{2}^{k-1}-x_{2}^{k} \bigr\Vert _{G_{0}}^{2}\bigr) \\& \qquad {}-\frac{(1-\alpha)\beta}{2}\bigl(3 \bigl\Vert A_{2}\bigl(x_{2}^{k}-x_{2}^{k+1} \bigr) \bigr\Vert ^{2}+ \bigl\Vert A_{2} \bigl(x_{2}^{k-1}-x_{2}^{k}\bigr) \bigr\Vert ^{2}\bigr), \end{aligned}$$

where the last inequality comes from the Cauchy-Schwartz inequality. Substituting the above inequality into the right-hand side of (30) and arranging terms, we get the assertion (28) immediately. □

Then, substituting (28) into the right-hand side of (26), we get the following main theorem, which provides a lower bound of as $\|w^{k}-\tilde{w}^{k}\|_{R}^{2}$, and the lower bound is composed of the term $\|Ax^{k+1}-b\|^{2}$, some terms in the form $\|w-w^{k+1}\| ^{2}-\|w-w^{k}\|^{2}$, and some others.

### Theorem 3.1

*Let* $\{(x^{k},\lambda^{k})\}=\{(x_{1}^{k},x_{2}^{k},\lambda ^{k})\}$ *be the sequence generated by the IPS*-*ADMM*. *Then we have*

31 $$\begin{aligned}& \bigl\Vert w^{k}-\tilde{w}^{k} \bigr\Vert _{R}^{2} \\& \quad \geq \bigl\Vert x_{1}^{k}-x_{1}^{k+1} \bigr\Vert _{P}^{2}+\alpha \bigl\Vert x_{2}^{k}-x_{2}^{k+1} \bigr\Vert _{G_{0}}^{2}-(1-\alpha)\beta \bigl\Vert A_{2} \bigl(x_{2}^{k}-x_{2}^{k+1}\bigr) \bigr\Vert ^{2} \\& \qquad {}+\frac {(1-r)^{2}}{1+r}\beta \bigl\Vert A_{2} \bigl(x_{2}^{k}-x_{2}^{k+1}\bigr) \bigr\Vert ^{2} +(2-r-s)\beta \bigl\Vert Ax^{k+1}-b \bigr\Vert ^{2} \\& \qquad {}+\frac{2(1-r)(1-s)}{1+r}\beta \bigl(Ax^{k}-b \bigr)^{\top}A_{2}\bigl(x_{2}^{k}-x_{2}^{k+1} \bigr) \\& \qquad {} +\frac{(1-r)\alpha}{1+r}\bigl( \bigl\Vert x_{2}^{k}-x_{2}^{k+1} \bigr\Vert _{G_{0}}^{2}- \bigl\Vert x_{2}^{k-1}-x_{2}^{k} \bigr\Vert _{G_{0}}^{2}\bigr) \\& \qquad {} -\frac{(1-r)(1-\alpha)\beta}{1+r}\bigl(3 \bigl\Vert A_{2} \bigl(x_{2}^{k}-x_{2}^{k+1}\bigr) \bigr\Vert ^{2}+ \bigl\Vert A_{2}\bigl(x_{2}^{k-1}-x_{2}^{k} \bigr) \bigr\Vert ^{2}\bigr). \end{aligned}$$

Now, let us rewrite all the terms on the right-hand side of (31) by some quadratic terms, and mainly deal with the term $\| x_{2}^{k}-x_{2}^{k+1}\|_{G_{0}}^{2}$ and the crossing term $(Ax^{k}-b)^{\top}A_{2}(x_{2}^{k}-x_{2}^{k+1})$. According to the simplicial partition $\mathcal{D}_{i}$ ($i=1,2,\ldots,5$) of the set $\mathcal{D}$ in (6), the following analysis is divided into five cases, which are discussed in the following five subsections.

### Case 1: $(r,s)\in\mathcal{D}_{1}$

#### Lemma 3.4

*For any fixed* $(r,s)\in\mathcal{D}_{1}$, *if* $\alpha >\alpha_{1}\doteq s+\frac{(1-s)^{2}}{2-r-s}$, *then there are constants* $C_{11},C_{12}>0$ *such that*

32 $$ \bigl\Vert w^{k}-\tilde{w}^{k} \bigr\Vert _{R}^{2}\geq \bigl\Vert x_{1}^{k}-x_{1}^{k+1} \bigr\Vert _{P}^{2}+C_{11}\beta \bigl\Vert A_{2}\bigl(x_{2}^{k}-x_{2}^{k+1} \bigr) \bigr\Vert ^{2}+C_{12}\beta \bigl\Vert Ax^{k+1}-b \bigr\Vert ^{2}. $$

*Furthermore*, $\alpha_{1}\in(\alpha_{0},1)$, *for any* $(r,s)\in\mathcal {D}_{1}$, *where* $\alpha_{0}$ *is defined in* (15).

#### Proof

We prove the assertion (32) from the definition of the matrix *R* directly. Define an auxiliary matrix $R_{0}$ as

$$R_{0}=\left ( \textstyle\begin{array}{@{}c@{\quad}c@{\quad}c@{}} P&0&0\\ 0&(\alpha-s)\beta A_{2}^{\top}A_{2}&-(1-s)A_{2}^{\top}\\ 0&-(1-s)A_{2}&\frac{2-(r+s)}{\beta}I_{m} \end{array}\displaystyle \right ). $$

By the expression of *R* in (25), we have

$$\begin{aligned} R(2:3,2:3) =&\left ( \textstyle\begin{array}{@{}c@{\quad}c@{}} \alpha G_{0}+(\alpha-s)\beta A_{2}^{\top}A_{2}&-(1-s)A_{2}^{\top}\\ -(1-s)A_{2}&\frac{2-(r+s)}{\beta}I_{m} \end{array}\displaystyle \right ) \\ =&\left ( \textstyle\begin{array}{@{}c@{\quad}c@{}} \alpha G_{0}&0\\ 0&0 \end{array}\displaystyle \right )+R_{0}(2:3,2:3) \\ \succeq& R_{0}(2:3,2:3) \\ =&\left ( \textstyle\begin{array}{@{}c@{\quad}c@{}} A_{2}^{\top}&0\\ 0&I_{m} \end{array}\displaystyle \right )\left ( \textstyle\begin{array}{@{}c@{\quad}c@{}} \beta(\alpha-s)I_{m}&-(1-s)I_{m}\\ -(1-s)I_{m}&\frac{2-r-s}{\beta}I_{m} \end{array}\displaystyle \right )\left ( \textstyle\begin{array}{@{}c@{\quad}c@{}} A_{2}&0\\ 0&I_{m} \end{array}\displaystyle \right ). \end{aligned}$$

Now, let us verify the positive definiteness of the matrix

$${S}\doteq \left ( \textstyle\begin{array}{@{}c@{\quad}c@{}} \beta(\alpha-s)I_{m}&-(1-s)I_{m}\\ -(1-s)I_{m}&\frac{2-r-s}{\beta}I_{m} \end{array}\displaystyle \right ), $$

which can be written as

$$\left ( \textstyle\begin{array}{@{}c@{\quad}c@{}} \beta(\alpha-s)&-(1-s)\\ -(1-s)&\frac{2-r-s}{\beta} \end{array}\displaystyle \right )\otimes I_{m}. $$

Obviously, when $\alpha>\alpha_{1}$, the above matrix is positive definite. Therefore, the matrix *S* is positive definite, and then the matrices *R* and $R_{0}$ are both positive definite by the full column rank of $A_{2}$ and the positive definiteness of *P*. By a manipulation, we get

$$\begin{aligned}& \bigl\Vert w^{k}-\tilde{w}^{k} \bigr\Vert _{R}^{2} \\& \quad \geq \bigl\Vert w^{k}-\tilde{w}^{k} \bigr\Vert _{R_{0}}^{2} \\& \quad = \bigl\Vert x_{1}^{k}-x_{1}^{k+1} \bigr\Vert _{P}^{2}+ \left \Vert \textstyle\begin{array}{@{}c@{}} A_{2}(x_{2}^{k}-x_{2}^{k+1})\\ Ax^{k+1}-b \end{array}\displaystyle \right \Vert _{\tilde{S}}^{2}, \end{aligned}$$

where

$$\tilde{S}=L^{\top}SL,\quad L=\left ( \textstyle\begin{array}{@{}c@{\quad}c@{}} I_{m}&0\\ \beta I_{m}&\beta I_{m} \end{array}\displaystyle \right ). $$

By the positive definiteness of the matrix *S*, we get the assertion (32). By the definitions of $\alpha_{0}$ and $\alpha_{1}$, we have

$$\alpha_{1}-\alpha_{0}=\frac{(1-r)^{2}}{2-r-s}>0, \quad \forall(r,s)\in\mathcal{D}_{1}. $$

Therefore, $\alpha_{1}>\alpha_{0}$, for any $(r,s)\in\mathcal{D}_{1}$. By some manipulations, we have

$$1-\alpha_{1}=\frac{(1-r)(1-s)}{2-r-s}>0,\quad \forall(r,s)\in \mathcal{D}_{1}. $$

Therefore, $\alpha_{1}\in(\alpha_{0},1)$, for any $(r,s)\in\mathcal{D}_{1}$. □

### Case 2: $(r,s)\in\mathcal{D}_{2}$

#### Lemma 3.5

*For any* $(r,s)\in\mathcal{D}_{2}$, *if* $\alpha>\alpha _{2}\doteq\frac{4-r-r^{2}}{5-3r}$, *then we have*

33 $$\begin{aligned}& \bigl\Vert w^{k}-\tilde{w}^{k} \bigr\Vert _{R}^{2} \\& \quad \geq \bigl\Vert x_{1}^{k}-x_{1}^{k+1} \bigr\Vert _{P}^{2}+C_{1}\beta \bigl\Vert A_{2}\bigl(x_{2}^{k}-x_{2}^{k+1} \bigr) \bigr\Vert ^{2} \\& \qquad {}+C_{2}\beta \bigl\Vert Ax^{k+1}-b \bigr\Vert ^{2}+C_{3}\bigl( \bigl\Vert x_{2}^{k}-x_{2}^{k+1} \bigr\Vert _{G_{0}}^{2}- \bigl\Vert x_{2}^{k-1}-x_{2}^{k} \bigr\Vert _{G_{0}}^{2}\bigr) \\& \qquad {}+C_{4}\beta\bigl( \bigl\Vert A_{2} \bigl(x_{2}^{k}-x_{2}^{k+1}\bigr) \bigr\Vert ^{2}- \bigl\Vert A_{2}\bigl(x_{2}^{k-1}-x_{2}^{k} \bigr) \bigr\Vert ^{2}\bigr), \end{aligned}$$

*where* $C_{2i}$ ($i=1,2,3,4$) *are four positive constants defined by*

$$\begin{aligned}& C_{21}=\frac{(5-3r)\alpha+ r^{2} + r - 4}{1+r},\qquad C_{22}=1-r, \\& C_{23}=\frac {(1-r)\alpha}{1+r},\qquad C_{24}=\frac{(1-r)(1-\alpha)}{1+r}. \end{aligned}$$

*Furthermore*, $\alpha_{2}\in(\alpha_{0},1)$, *for any* $(r,s)\in\mathcal{D}_{2}$.

#### Proof

Setting $s=1$ in (31), we have

$$\begin{aligned}& \bigl\Vert w^{k}-\tilde{w}^{k} \bigr\Vert _{R}^{2} \\& \quad \geq \bigl\Vert x_{1}^{k}-x_{1}^{k+1} \bigr\Vert _{P}^{2}+\alpha \bigl\Vert x_{2}^{k}-x_{2}^{k+1} \bigr\Vert _{G_{0}}^{2}-(1-\alpha)\beta \bigl\Vert A_{2} \bigl(x_{2}^{k}-x_{2}^{k+1}\bigr) \bigr\Vert ^{2} \\& \qquad {} +\frac {(1-r)^{2}}{1+r}\beta \bigl\Vert A_{2} \bigl(x_{2}^{k}-x_{2}^{k+1}\bigr) \bigr\Vert ^{2}+(1-r)\beta \bigl\Vert Ax^{k+1}-b \bigr\Vert ^{2} \\& \qquad {}+\frac{(1-r)\alpha}{1+r}\bigl( \bigl\Vert x_{2}^{k}-x_{2}^{k+1} \bigr\Vert _{G_{0}}^{2}- \bigl\Vert x_{2}^{k-1}-x_{2}^{k} \bigr\Vert _{G_{0}}^{2}\bigr) \\& \qquad {} -\frac{(1-r)(1-\alpha)\beta}{1+r}\bigl(3 \bigl\Vert A_{2} \bigl(x_{2}^{k}-x_{2}^{k+1}\bigr) \bigr\Vert ^{2}+ \bigl\Vert A_{2}\bigl(x_{2}^{k-1}-x_{2}^{k} \bigr) \bigr\Vert ^{2}\bigr) \\& \quad \geq\frac{(5-3r)\alpha+ r^{2} + r - 4}{1+r}\beta \bigl\Vert A_{2} \bigl(x_{2}^{k}-x_{2}^{k+1}\bigr) \bigr\Vert ^{2} \\& \qquad {} +(1-r)\beta \bigl\Vert Ax^{k+1}-b \bigr\Vert ^{2}+\frac{(1-r)\alpha}{1+r}\bigl( \bigl\Vert x_{2}^{k}-x_{2}^{k+1} \bigr\Vert _{G_{0}}^{2}- \bigl\Vert x_{2}^{k-1}-x_{2}^{k} \bigr\Vert _{G_{0}}^{2}\bigr) \\& \qquad {} +\frac{(1-r)(1-\alpha)\beta}{1+r}\bigl( \bigl\Vert A_{2} \bigl(x_{2}^{k}-x_{2}^{k+1}\bigr) \bigr\Vert ^{2}- \bigl\Vert A_{2}\bigl(x_{2}^{k-1}-x_{2}^{k} \bigr) \bigr\Vert ^{2}\bigr), \end{aligned}$$

which proves (33). From $\alpha>\alpha_{2}$, it is obvious that $C_{21}>0$, and from $r\in(-1,1)$, $\alpha\in(\alpha_{2},1)$, we have $C_{22},C_{23},C_{24}>0$. By the definition of $\alpha_{2}$, we get

$$\alpha_{2}-\alpha_{0}=\frac{2(1-r)(2-r)}{5-3r}>0,\quad \forall(r,s)\in\mathcal{D}_{2}. $$

Therefore, $\alpha_{2}>\alpha_{0}$, for any $(r,s)\in\mathcal{D}_{2}$. By some manipulations, we have

$$1-\alpha_{2}=\frac{(r - 1)^{2}}{5-3r}>0,\quad \forall(r,s)\in \mathcal{D}_{2}. $$

Therefore, $\alpha_{2}\in(\alpha_{0},1)$, for any $(r,s)\in\mathcal{D}_{2}$. □

#### Remark 3.3

**Figure 1:**
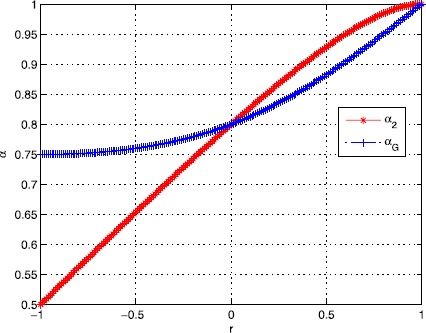
**The curves of** $\pmb{\alpha_{2}}$ **and** $\pmb{\alpha_{G}}$ **in** $\pmb{r\in(-1,1)}$ **.**

For any $(r,s)\in\mathcal{D}_{2}$, Gao *et al.* [21] have proved that $\alpha_{G}\doteq\frac{r^{2}-r+4}{r^{2}-2r+5}$ is a lower bound of *α*. The curves of $\alpha_{2}$ and $\alpha_{G}$ with $r\in(-1,1)$ are drawn in Figure 1, from which we have $\alpha_{2}<\alpha_{G}$ if $r\in(-1,0)$, and $\alpha_{2}>\alpha_{G}$ if $r\in(0,1)$. Therefore, compared with that in [21], the feasible set of *τ* in this paper is expanded if $r\in(-1,0)$, and is shrunk if $r\in(0,1)$. However, Gao *et al.* only established the worst-case convergence rate of the IPS-ADMM using the criterion (18), and we shall prove the worst-case convergence rate of the IPS-ADMM using the more reasonable criterion (19); see the following Theorem 3.4.

### Case 3: $(r,s)\in\mathcal{D}_{3}$

#### Lemma 3.6

*For any* $(r,s)\in\mathcal{D}_{3}$, *if* $\alpha>\alpha _{3}\doteq\frac{7s^{2}-22s+23}{5s^{2}-20s+25}$, *then we have*

34 $$\begin{aligned}& \bigl\Vert w^{k}-\tilde{w}^{k} \bigr\Vert _{R}^{2} \\& \quad \geq \bigl\Vert x_{1}^{k}-x_{1}^{k+1} \bigr\Vert _{P}^{2}+C_{30}\beta\bigl( \bigl\Vert Ax^{k+1}-b \bigr\Vert ^{2}- \bigl\Vert Ax^{k}-b \bigr\Vert ^{2}\bigr)+C_{31}\beta \bigl\Vert A_{2}\bigl(x_{2}^{k}-x_{2}^{k+1} \bigr) \bigr\Vert ^{2} \\& \qquad{} +C_{32}\beta \bigl\Vert Ax^{k+1}-b \bigr\Vert ^{2}+C_{33}\bigl( \bigl\Vert x_{2}^{k}-x_{2}^{k+1} \bigr\Vert _{G_{0}}^{2}- \bigl\Vert x_{2}^{k-1}-x_{2}^{k} \bigr\Vert _{G_{0}}^{2}\bigr) \\& \qquad {} +C_{34}\beta\bigl( \bigl\Vert A_{2} \bigl(x_{2}^{k}-x_{2}^{k+1}\bigr) \bigr\Vert ^{2}- \bigl\Vert A_{2}\bigl(x_{2}^{k-1}-x_{2}^{k} \bigr) \bigr\Vert ^{2}\bigr), \end{aligned}$$

*where* $C_{3i}$ ($i=0,1,2,3,4$) *are five positive constants defined by*

$$\begin{aligned}& C_{30}=T_{1}-s,\qquad C_{31}=1- \frac{(1-s)^{2}}{T_{1}-s}-5(1-\alpha),\qquad C_{32}=2-T_{1}, \\& C_{33}=\alpha,\qquad C_{34}=1-\alpha,\quad T_{1}= \frac{1}{3}\bigl(s^{2}-s+5\bigr). \end{aligned}$$

*Furthermore*, $\alpha_{3}\in(\alpha_{0},1)$, *for any* $(r,s)\in\mathcal{D}_{3}$.

#### Proof

By the Cauchy-Schwartz inequality, we have

$$2(1-s)\beta\bigl(Ax^{k}-b\bigr)^{\top}A_{2} \bigl(x_{2}^{k}-x_{2}^{k+1}\bigr) \geq-(T_{1}-s)\beta \bigl\Vert Ax^{k}-b \bigr\Vert ^{2}-\frac{(1-s)^{2}}{T_{1}-s} \bigl\Vert A_{2} \bigl(x_{2}^{k}-x_{2}^{k+1}\bigr) \bigr\Vert ^{2}. $$

Then, substituting the above inequality into the right-hand side of (31), we get

$$\begin{aligned}& \bigl\Vert w^{k}-\tilde{w}^{k} \bigr\Vert _{R}^{2} \\& \quad \geq \bigl\Vert x_{1}^{k}-x_{1}^{k+1} \bigr\Vert _{P}^{2}+(T_{1}-s)\beta\bigl( \bigl\Vert Ax^{k+1}-b \bigr\Vert ^{2}- \bigl\Vert Ax^{k}-b \bigr\Vert ^{2}\bigr) \\& \qquad {} +\biggl(1-\frac{(1-s)^{2}}{T_{1}-s}-5(1-\alpha)\biggr)\beta \bigl\Vert A_{2}\bigl(x_{2}^{k}-x_{2}^{k+1} \bigr) \bigr\Vert ^{2} \\& \qquad {} +(2-T_{1})\beta \bigl\Vert Ax^{k+1}-b \bigr\Vert ^{2}+\alpha\bigl( \bigl\Vert x_{2}^{k}-x_{2}^{k+1} \bigr\Vert _{G_{0}}^{2}- \bigl\Vert x_{2}^{k-1}-x_{2}^{k} \bigr\Vert _{G_{0}}^{2}\bigr) \\& \qquad {} +(1-\alpha)\beta\bigl( \bigl\Vert A_{2} \bigl(x_{2}^{k}-x_{2}^{k+1}\bigr) \bigr\Vert ^{2}- \bigl\Vert A_{2}\bigl(x_{2}^{k-1}-x_{2}^{k} \bigr) \bigr\Vert ^{2}\bigr), \end{aligned}$$

which proves (34). From $\alpha\in(\alpha_{3},1)$, it is obvious that

$$\begin{aligned}& C_{30}=\frac{1}{3}\bigl[(s-2)^{2}+1\bigr]> \frac{1}{3}, \\& C_{31}=\frac{(5s^{2} - 20s + 25)\alpha- 7s^{2} + 22s - 23}{s^{2} - 4s + 5}>0, \\& C_{32}=\frac{1}{3}\bigl(1+s-s^{2}\bigr)>0, \\& C_{33}=\alpha>0, \qquad C_{34}=1-\alpha>0. \end{aligned}$$

Furthermore, by some manipulations, $\forall(r,s)\in\mathcal{D}_{3}$, we have

$$\begin{aligned}& \alpha_{3}-\alpha_{0}=\alpha_{3}= \frac{7s^{2}-22s+23}{5(s^{2}-4s+5)}>0, \\& 1-\alpha_{3}=\frac{-2(s-\frac{1-\sqrt{5}}{2})(s-\frac{1+\sqrt {5}}{2})}{5(s^{2}-4s+5)}>0. \end{aligned}$$

Therefore, $\alpha_{3}\in(\alpha_{0},1)$, for any $(r,s)\in\mathcal{D}_{3}$. □

### Case 4: $(r,s)\in\mathcal{D}_{4}$

#### Lemma 3.7

*For any* $(r,s)\in\mathcal{D}_{4}$, *if* $\alpha>\alpha _{4}\doteq\frac{r^{3} + r^{2} - r - 5}{3r^{2} - 2r - 5}$, *then we have*

35 $$\begin{aligned}& \bigl\Vert w^{k}-\tilde{w}^{k} \bigr\Vert _{R}^{2} \\& \quad \geq \bigl\Vert x_{1}^{k}-x_{1}^{k+1} \bigr\Vert _{P}^{2}+C_{40}\beta\bigl( \bigl\Vert Ax^{k+1}-b \bigr\Vert ^{2}- \bigl\Vert Ax^{k}-b \bigr\Vert ^{2}\bigr)+C_{41}\beta \bigl\Vert A_{2}\bigl(x_{2}^{k}-x_{2}^{k+1} \bigr) \bigr\Vert ^{2} \\& \qquad {} +C_{42}\beta \bigl\Vert Ax^{k+1}-b \bigr\Vert ^{2}+C_{43}\bigl( \bigl\Vert x_{2}^{k}-x_{2}^{k+1} \bigr\Vert _{G_{0}}^{2}- \bigl\Vert x_{2}^{k-1}-x_{2}^{k} \bigr\Vert _{G_{0}}^{2}\bigr) \\& \qquad {}+C_{44}\beta\bigl( \bigl\Vert A_{2} \bigl(x_{2}^{k}-x_{2}^{k+1}\bigr) \bigr\Vert ^{2}- \bigl\Vert A_{2}\bigl(x_{2}^{k-1}-x_{2}^{k} \bigr) \bigr\Vert ^{2}\bigr), \end{aligned}$$

*where* $C_{4i}$ ($i=0,1,2,3,4$) *are five positive constants defined by*

$$\begin{aligned}& C_{40}=T_{2}-(r+s),\qquad C_{41}=r \frac{(1-r)^{2}}{(1+r)^{2}}-4(1-\alpha)\frac {1-r}{1+r}+(\alpha-1), \\& C_{42}=2-T_{2},\qquad C_{43}=\alpha \frac{1-r}{1+r},\qquad C_{44}=(1-\alpha)\frac {1-r}{1+r},\quad T_{2}=r+s+(1-s)^{2}. \end{aligned}$$

*Furthermore*, $\alpha_{4}\in(\alpha_{0},1)$, *for any* $(r,s)\in\mathcal{D}_{4}$.

#### Proof

By the Cauchy-Schwartz inequality, we have

$$\begin{aligned}& 2\frac{(1-r)(1-s)}{1+r}\beta\bigl(Ax^{k}-b\bigr)^{\top}A_{2}\bigl(x_{2}^{k}-x_{2}^{k+1} \bigr) \\& \quad \geq-\bigl[T_{2}-(r+s)\bigr]\beta \bigl\Vert Ax^{k}-b \bigr\Vert ^{2}-\frac {(1-r)^{2}(1-s)^{2}}{(1+r)^{2}[T_{2}-(r+s)]} \bigl\Vert A_{2}\bigl(x_{2}^{k}-x_{2}^{k+1} \bigr) \bigr\Vert ^{2}. \end{aligned}$$

Then, substituting the above inequality into the right-hand side of (31), we get

$$\begin{aligned}& \bigl\Vert w^{k}-\tilde{w}^{k} \bigr\Vert _{R}^{2} \\& \quad \geq \bigl\Vert x_{1}^{k}-x_{1}^{k+1} \bigr\Vert _{P}^{2}+\bigl[T_{2}-(r+s)\bigr]\beta \bigl( \bigl\Vert Ax^{k+1}-b \bigr\Vert ^{2}- \bigl\Vert Ax^{k}-b \bigr\Vert ^{2}\bigr) \\& \qquad {}+\biggl(r\frac{(1-r)^{2}}{(1+r)^{2}}-4(1-\alpha)\frac{1-r}{1+r}+(\alpha -1) \biggr)\beta \bigl\Vert A_{2}\bigl(x_{2}^{k}-x_{2}^{k+1} \bigr) \bigr\Vert ^{2} \\& \qquad {}+(2-T_{2})\beta \bigl\Vert Ax^{k+1}-b \bigr\Vert ^{2}+\alpha\frac{1-r}{1+r}\bigl( \bigl\Vert x_{2}^{k}-x_{2}^{k+1} \bigr\Vert _{G_{0}}^{2}- \bigl\Vert x_{2}^{k-1}-x_{2}^{k} \bigr\Vert _{G_{0}}^{2}\bigr) \\& \qquad {}+(1-\alpha)\frac{1-r}{1+r}\beta\bigl( \bigl\Vert A_{2} \bigl(x_{2}^{k}-x_{2}^{k+1}\bigr) \bigr\Vert ^{2}- \bigl\Vert A_{2}\bigl(x_{2}^{k-1}-x_{2}^{k} \bigr) \bigr\Vert ^{2}\bigr), \end{aligned}$$

which proves (35). From the definition of $T_{2}$, $\alpha\in (\alpha_{4},1)$, $(r,s)\in\mathcal{D}_{4}$, it is easy to verify that $C_{40},C_{42},C_{43},C_{44}>0$. From the definition of $C_{41}$, we get

$$C_{41}=\frac{(r+1)(5-3r)\alpha+ r^{3} + r^{2} - r - 5}{(r+1)^{2}}>0,\quad \forall \alpha>\alpha_{4}. $$

Furthermore, by some manipulations, $\forall(r,s)\in\mathcal{D}_{3}$, we have

$$\begin{aligned}& \alpha_{4}-\alpha_{0}=\frac{(1-r)[2(1-r^{2})+r+3]}{(1+ r)(5-3r)}>0, \\& 1-\alpha_{4}=\frac{r(r - 1)^{2}}{(1+ r)(5-3r)}>0. \end{aligned}$$

Therefore, $\alpha_{4}\in(\alpha_{0},1)$, for any $(r,s)\in\mathcal{D}_{4}$. □

### Case 5: $(r,s)\in\mathcal{D}_{5}$

#### Lemma 3.8

*For any* $(r,s)\in\mathcal{D}_{5}$, *if* $\alpha>\alpha _{5}\doteq\frac{(r^{2} + r - 4)s^{2} -( r^{2} +4r - 9)s - (r-1)^{2}}{s(2-s)(5-3r)}$, *then we have*

36 $$\begin{aligned}& \bigl\Vert w^{k}-\tilde{w}^{k} \bigr\Vert _{R}^{2} \\& \quad \geq \bigl\Vert x_{1}^{k}-x_{1}^{k+1} \bigr\Vert _{P}^{2}+ C_{0}\beta\bigl( \bigl\Vert Ax^{k+1}-b \bigr\Vert ^{2}- \bigl\Vert Ax^{k}-b \bigr\Vert ^{2}\bigr)+C_{1}\beta \bigl\Vert A_{2}\bigl(x_{2}^{k}-x_{2}^{k+1} \bigr) \bigr\Vert ^{2} \\& \qquad {} +C_{2}\beta \bigl\Vert Ax^{k+1}-b \bigr\Vert ^{2}+C_{3}\bigl( \bigl\Vert x_{2}^{k}-x_{2}^{k+1} \bigr\Vert _{G_{0}}^{2}- \bigl\Vert x_{2}^{k-1}-x_{2}^{k} \bigr\Vert _{G_{0}}^{2}\bigr) \\& \qquad {} +C_{4}\beta\bigl( \bigl\Vert A_{2} \bigl(x_{2}^{k}-x_{2}^{k+1}\bigr) \bigr\Vert ^{2}- \bigl\Vert A_{2}\bigl(x_{2}^{k-1}-x_{2}^{k} \bigr) \bigr\Vert ^{2}\bigr), \end{aligned}$$

*where* $C_{5i}$ ($i=0,1,2,3,4$) *are five positive constants defined by*

$$\begin{aligned}& C_{50}=\frac{(s^{2}-s)(2-s)}{1+r},\qquad C_{51}= \frac {(1-r)^{2}(1+s-s^{2})}{s(1+r)(2-s)}-4(1-\alpha)\frac{1-r}{1+r}+(\alpha-1), \\& C_{52}=2-T_{3},\qquad C_{53}=\alpha \frac{1-r}{1+r}, \\& C_{54}=(1-\alpha)\frac {1-r}{1+r},\quad T_{3}=r+s+\frac{(s^{2}-s)(2-s)}{1+r}. \end{aligned}$$

*Furthermore*, $\alpha_{5}\in(\alpha_{0},1)$, *for any* $(r,s)\in\mathcal{D}_{5}$.

#### Proof

By the Cauchy-Schwartz inequality, we have

$$\begin{aligned}& 2\frac{(1-r)(1-s)}{1+r}\beta\bigl(Ax^{k}-b\bigr)^{\top}A_{2}\bigl(x_{2}^{k}-x_{2}^{k+1} \bigr) \\& \quad \geq-\bigl[T_{3}-(r+s)\bigr]\beta \bigl\Vert Ax^{k}-b \bigr\Vert ^{2}-\frac {(1-r)^{2}(1-s)^{2}}{(1+r)^{2}[T_{3}-(r+s)]} \bigl\Vert A_{2}\bigl(x_{2}^{k}-x_{2}^{k+1} \bigr) \bigr\Vert ^{2}. \end{aligned}$$

Then, substituting the above inequality into the right-hand side of (31), we get

$$\begin{aligned}& \bigl\Vert w^{k}-\tilde{w}^{k} \bigr\Vert _{R}^{2} \\& \quad \geq \bigl\Vert x_{1}^{k}-x_{1}^{k+1} \bigr\Vert _{P}^{2}+\bigl[T_{3}-(r+s)\bigr]\beta \bigl( \bigl\Vert Ax^{k+1}-b \bigr\Vert ^{2}- \bigl\Vert Ax^{k}-b \bigr\Vert ^{2}\bigr) \\& \qquad {}+\biggl(\frac{(1-r)^{2}(1+s-s^{2})}{s(1+r)(2-s)}-4(1-\alpha)\frac {1-r}{1+r}+(\alpha-1) \biggr)\beta \bigl\Vert A_{2}\bigl(x_{2}^{k}-x_{2}^{k+1} \bigr) \bigr\Vert ^{2} \\& \qquad {}+(2-T_{3})\beta \bigl\Vert Ax^{k+1}-b \bigr\Vert ^{2}+\alpha\frac{1-r}{1+r}\bigl( \bigl\Vert x_{2}^{k}-x_{2}^{k+1} \bigr\Vert _{G_{0}}^{2}- \bigl\Vert x_{2}^{k-1}-x_{2}^{k} \bigr\Vert _{G_{0}}^{2}\bigr) \\& \qquad {}+(1-\alpha)\frac{1-r}{1+r}\beta\bigl( \bigl\Vert A_{2} \bigl(x_{2}^{k}-x_{2}^{k+1}\bigr) \bigr\Vert ^{2}- \bigl\Vert A_{2}\bigl(x_{2}^{k-1}-x_{2}^{k} \bigr) \bigr\Vert ^{2}\bigr), \end{aligned}$$

which proves (36). From the definition of $T_{3}$, $\alpha\in (\alpha_{5},1)$, $(r,s)\in\mathcal{D}_{5}$, it is easy to verify that $C_{50},C_{52},C_{53},C_{54}>0$. From the definition of $C_{51}$, for any $(r,s)\in\mathcal{D}_{5}$, we get

$$C_{51}=\frac{s(2-s)(5-3r)\alpha-(r^{2} + r - 4)s^{2} + (r^{2}+ 4r - 9)s + (r-1)^{2}}{s(2-s)(r + 1)}>0,\quad \forall\alpha>\alpha_{5}. $$

By the definition of $\alpha_{5}$, for any $(r,s)\in\mathcal{D}_{5}$, we have

$$\begin{aligned}& \alpha_{5}-\alpha_{0} \\& \quad =\frac{(1-r)[2(r - 2)s^{2} + (9 - 5r)s + r - 1]}{s(5-3r)(2-s)} \\& \quad \geq\frac{(1-r)[2(r - 2)(1+r+s) + (9 - 5r)s + r - 1]}{s(5-3r)(2-s)} \\& \quad =\frac{(1-r)[5(s-1)+r(2r-3s-1)]}{s(5-3r)(2-s)} \\& \quad >0, \end{aligned}$$

where the first inequality follows from $s^{2}<1+r+s$, and the second inequality comes from $r<0$, $s\in(1,\frac{1+\sqrt{5}}{2})$, $r<\frac{3s+1}{2}$. By some manipulations, we obtain

$$1-\alpha_{5}=\frac{-(r - 1)^{2}(s-\frac{1-\sqrt{5}}{2})(s-\frac{1+\sqrt {5}}{2})}{s(5-3r)(2-s)}>0,\quad \forall(r,s)\in \mathcal{D}_{5}. $$

Therefore, $\alpha_{5}\in(\alpha_{0},1)$, for any $(r,s)\in\mathcal{D}_{5}$. □

In the remainder of this section, we shall establish the convergence results of the sequence generated by the IPS-ADMM. First, based on (24) and Lemmas 3.4-3.8, we can get the following theorem.

#### Theorem 3.2

*Let* $\{(x^{k},\lambda^{k})\}=\{(x_{1}^{k},x_{2}^{k},\lambda ^{k})\}$ *be the sequence generated by the IPS*-*ADMM*. *Then*, *for any* $(r,s)\in\mathcal{D}$, $\alpha\in(c(r,s),1)$, *where* $c(r,s)$ *is defined in* (8), *we have*

37 $$\begin{aligned}& \theta\bigl(x^{k+1}\bigr)-\theta(x)+\bigl({x}^{k+1}-x \bigr)^{\top}\bigl(-A^{\top}\lambda\bigr) \\& \quad \leq\frac{1}{2}\bigl( \bigl\Vert w-w^{k} \bigr\Vert _{H}^{2}- \bigl\Vert w-w^{k+1} \bigr\Vert ^{2}_{H}\bigr)-\frac{1}{2} \bigl\Vert x_{1}^{k}-x_{1}^{k+1} \bigr\Vert _{P}^{2} \\& \qquad {}-\frac{C_{0}}{2}\beta\bigl( \bigl\Vert Ax^{k+1}-b \bigr\Vert ^{2}- \bigl\Vert Ax^{k}-b \bigr\Vert ^{2}\bigr) \\& \qquad {}-\frac{C_{1}}{2}\beta \bigl\Vert A_{2} \bigl(x_{2}^{k}-x_{2}^{k+1}\bigr) \bigr\Vert ^{2}-\frac{C_{2}}{2}\beta \bigl\Vert Ax^{k+1}-b \bigr\Vert ^{2} \\& \qquad {}-\frac{C_{3}}{2}\bigl( \bigl\Vert x_{2}^{k}-x_{2}^{k+1} \bigr\Vert _{G_{0}}^{2}- \bigl\Vert x_{2}^{k-1}-x_{2}^{k} \bigr\Vert _{G_{0}}^{2}\bigr) \\& \qquad {}-\frac{C_{4}}{2}\beta\bigl( \bigl\Vert A_{2} \bigl(x_{2}^{k}-x_{2}^{k+1}\bigr) \bigr\Vert ^{2}- \bigl\Vert A_{2}\bigl(x_{2}^{k-1}-x_{2}^{k} \bigr) \bigr\Vert ^{2}\bigr), \end{aligned}$$

*where* $(x_{1},x_{2},\lambda)\in\mathcal{R}^{m+n_{1}+n_{2}}$ *satisfies* $A_{1}x_{1}+A_{2}x_{2}=b$, $C_{0},C_{3},C_{4}\geq0$, $C_{1},C_{2}>0$ *with* $C_{j}=C_{ij}$ *if* $(r,s)\in\mathcal{D}_{i}$, $i=1,2,3,4,5$, $j=0,1,2,3,4$.

With the above theorems in hand, now we are ready to prove the global convergence of the IPS-ADMM.

#### Theorem 3.3

*Let* $\{(x^{k},\lambda^{k})\}=\{(x_{1}^{k},x_{2}^{k},\lambda ^{k})\}$ *be the sequence generated by the IPS*-*ADMM*. *Then*, *if* $A_{1}$, $A_{2}$ *are both full column rank*, *the sequence* $\{(x^{k},\lambda^{k})\}$ *is bounded and converges to a point* $(x^{\infty},\lambda^{\infty})\in\mathcal{W}^{*}$.

#### Proof

Choose an arbitrary $(x_{1}^{*},x_{2}^{*},\lambda^{*})\in\mathcal {W}^{*}$ and setting $x_{1}=x_{1}^{*}$, $x_{2}=x_{2}^{*}$, $\lambda=\lambda^{*}$ in (37), we get

$$\begin{aligned}& \theta\bigl(x^{k+1}\bigr)-\theta\bigl(x^{*}\bigr)+\bigl({x}^{k+1}-x^{*} \bigr)^{\top}\bigl(-A^{\top}\lambda^{*}\bigr) \\& \quad \leq\frac{1}{2}\bigl( \bigl\Vert w^{*}-w^{k} \bigr\Vert _{H}^{2}- \bigl\Vert w^{*}-w^{k+1} \bigr\Vert ^{2}_{H}\bigr)-\frac{1}{2} \bigl\Vert x_{1}^{k}-x_{1}^{k+1} \bigr\Vert _{P}^{2} \\& \qquad {}-\frac{ C_{0}}{2}\beta\bigl( \bigl\Vert Ax^{k+1}-b \bigr\Vert ^{2}- \bigl\Vert Ax^{k}-b \bigr\Vert ^{2}\bigr) \\& \qquad {} -\frac{C_{1}}{2}\beta \bigl\Vert A_{2} \bigl(x_{2}^{k}-x_{2}^{k+1}\bigr) \bigr\Vert ^{2}-\frac{C_{2}}{2}\beta \bigl\Vert Ax^{k+1}-b \bigr\Vert ^{2} \\& \qquad {}-\frac{C_{3}}{2}\bigl( \bigl\Vert x_{2}^{k}-x_{2}^{k+1} \bigr\Vert _{G_{0}}^{2}- \bigl\Vert x_{2}^{k-1}-x_{2}^{k} \bigr\Vert _{G_{0}}^{2}\bigr) \\& \qquad {}-\frac{C_{4}}{2}\beta\bigl( \bigl\Vert A_{2} \bigl(x_{2}^{k}-x_{2}^{k+1}\bigr) \bigr\Vert ^{2}- \bigl\Vert A_{2}\bigl(x_{2}^{k-1}-x_{2}^{k} \bigr) \bigr\Vert ^{2}\bigr). \end{aligned}$$

Then, from $x\in\mathcal{X}_{1}\times\mathcal{X}_{2}$, $(x_{1}^{*},x_{2}^{*},\lambda ^{*})\in\mathcal{W}^{*}$ and (23), we have

38 $$\begin{aligned}& \bigl\Vert w^{k+1}-w^{*} \bigr\Vert ^{2}_{H}+C_{0} \beta \bigl\Vert Ax^{k+1}-b \bigr\Vert ^{2}+C_{3} \bigl\Vert x_{2}^{k}-x_{2}^{k+1} \bigr\Vert _{G_{0}}^{2}+C_{4}\beta \bigl\Vert A_{2}\bigl(x_{2}^{k}-x_{2}^{k+1} \bigr) \bigr\Vert ^{2} \\& \quad \leq \bigl\Vert w^{k}-w^{*} \bigr\Vert ^{2}_{H}+C_{0} \beta \bigl\Vert Ax^{k}-b \bigr\Vert ^{2}+C_{3} \bigl\Vert x_{2}^{k-1}-x_{2}^{k} \bigr\Vert _{G_{0}}^{2}+C_{4}\beta \bigl\Vert A_{2}\bigl(x_{2}^{k-1}-x_{2}^{k} \bigr) \bigr\Vert ^{2} \\& \qquad {} - \bigl\Vert x_{1}^{k}-x_{1}^{k+1} \bigr\Vert _{P}^{2}-C_{1}\beta \bigl\Vert A_{2}\bigl(x_{2}^{k}-x_{2}^{k+1} \bigr) \bigr\Vert ^{2}-C_{2}\beta \bigl\Vert Ax^{k+1}-b \bigr\Vert ^{2}, \end{aligned}$$

which together with $C_{0},C_{3},C_{4}\geq0$, $C_{1},C_{2}>0$, $H,G_{0}\succ0$ implies that

$$\begin{aligned}& \sum_{k=1}^{\infty}\bigl( \bigl\Vert x_{1}^{k}-x_{1}^{k+1} \bigr\Vert _{P}^{2}+C_{1}\beta \bigl\Vert A_{2} \bigl(x_{2}^{k}-x_{2}^{k+1}\bigr) \bigr\Vert ^{2}+C_{2}\beta \bigl\Vert Ax^{k+1}-b \bigr\Vert ^{2}\bigr) \\& \quad \leq \bigl\Vert w^{1}-w^{*} \bigr\Vert ^{2}_{H}+C_{0} \beta \bigl\Vert Ax^{1}-b \bigr\Vert ^{2}+C_{3} \bigl\Vert x_{2}^{0}-x_{2}^{1} \bigr\Vert _{G_{0}}^{2}+C_{4}\beta \bigl\Vert A_{2}\bigl(x_{2}^{0}-x_{2}^{1} \bigr) \bigr\Vert ^{2}< +\infty. \end{aligned}$$

This, the full column rank of $A_{2}$, and the positive definiteness of *P* indicate that

39 $$ \lim_{k\rightarrow\infty} \bigl\Vert x^{k}-x^{k+1} \bigr\Vert =\lim_{k\rightarrow\infty} \bigl\Vert Ax^{k+1}-b \bigr\Vert =0. $$

Furthermore, it follows from (38) that the sequences $\{w^{k}\} $ and $\{Ax^{k}-b\}$ are both bounded. Therefore, $\{w^{k}\}$ has at least one cluster point, saying $w^{\infty}$, and suppose that the subsequence $\{w^{k_{i}}\}$ converges to $w^{\infty}$. Then, taking the limits on both sides of (21) along the subsequence $\{w^{k_{i}}\}$ and using (39), we have

$$\theta(x)-\theta\bigl({x}^{\infty}\bigr)+\bigl(w-{w}^{\infty}\bigr)^{\top}F\bigl({w}^{\infty}\bigr)\geq0,\quad \forall w\in \mathcal{W}. $$

Therefore, $w^{\infty}\in\mathcal{W}^{*}$.

Hence, replacing $w^{*}$ by $w^{\infty}$ in (38), we get

$$\begin{aligned}& \bigl\Vert w^{k+1}-w^{\infty}\bigr\Vert ^{2}_{H}+C_{0} \beta \bigl\Vert Ax^{k+1}-b \bigr\Vert ^{2}+C_{3} \bigl\Vert x_{2}^{k}-x_{2}^{k+1} \bigr\Vert _{G_{0}}^{2}+C_{4}\beta \bigl\Vert A_{2}\bigl(x_{2}^{k}-x_{2}^{k+1} \bigr) \bigr\Vert ^{2} \\& \quad \leq \bigl\Vert w^{k}-w^{\infty}\bigr\Vert ^{2}_{H}+C_{0}\beta \bigl\Vert Ax^{k}-b \bigr\Vert ^{2}+C_{3} \bigl\Vert x_{2}^{k-1}-x_{2}^{k} \bigr\Vert _{G_{0}}^{2}+C_{4}\beta \bigl\Vert A_{2} \bigl(x_{2}^{k-1}-x_{2}^{k}\bigr) \bigr\Vert ^{2}. \end{aligned}$$

From (39), we see that, for any given $\varepsilon>0$, there exists $l_{0}>0$, such that

$$C_{0}\beta \bigl\Vert Ax^{k}-b \bigr\Vert ^{2}+C_{3} \bigl\Vert x_{2}^{k-1}-x_{2}^{k} \bigr\Vert _{G_{0}}^{2}+C_{4}\beta \bigl\Vert A_{2}\bigl(x_{2}^{k-1}-x_{2}^{k} \bigr) \bigr\Vert ^{2}< \frac{\varepsilon}{2},\quad \forall k\geq l_{0}. $$

Since $w^{k_{i}}\rightarrow w^{\infty}$ for $i\rightarrow\infty$, there exists $k_{l}>l_{0}$, such that

$$\bigl\Vert w^{k_{l}}-w^{\infty}\bigr\Vert ^{2}_{H}< \frac{\varepsilon}{2}. $$

Then the above three inequalities lead, for any $k>k_{l}$, to

$$\begin{aligned}& \bigl\Vert w^{k}-w^{\infty}\bigr\Vert ^{2}_{H} \\& \quad \leq \bigl\Vert w^{k_{l}}-w^{\infty}\bigr\Vert ^{2}_{H}+C_{0}\beta \bigl\Vert Ax^{k_{l}}-b \bigr\Vert ^{2}+C_{3} \bigl\Vert x_{2}^{k_{l}-1}-x_{2}^{k_{l}} \bigr\Vert _{G_{0}}^{2}+C_{4}\beta \bigl\Vert A_{2} \bigl(x_{2}^{{k_{l}}-1}-x_{2}^{k_{l}}\bigr) \bigr\Vert ^{2} \\& \quad < \varepsilon. \end{aligned}$$

Therefore the whole sequence $\{w^{k}\}$ converges to the $w^{\infty}$. The proof is completed. □

Now, we are going to prove the worst-case $\mathcal{O}(1/t)$ convergence rate in an ergodic sense of the IPS-ADMM.

#### Theorem 3.4

*Let* $\{(x^{k},\lambda^{k})\}=\{(x_{1}^{k},x_{2}^{k},\lambda ^{k})\}$ *be the sequence generated by the IPS*-*ADMM*, *and let*

$${x}_{t}=\frac{1}{t}\sum_{k=1}^{t}x^{k+1}, $$

*where* *t* *is a positive integer*. *Then*,

40 $$ \theta(x_{t})-\theta\bigl(x^{*}\bigr)+ \bigl(x_{t}-x^{*}\bigr)^{\top}\bigl(-A^{\top}\lambda ^{*} \bigr)+\frac{C_{2}}{2}\beta\|Ax_{t}-b\|^{2}\leq \frac{D}{t}, $$

*where* $(x^{*},\lambda^{*})\in\mathcal{W}^{*}$, *and* *D* *is a constant defined by*

41 $$ D=\frac{1}{2} \bigl\Vert v^{1}-v^{*} \bigr\Vert _{H}^{2}+\frac{C_{0}}{2}\beta \bigl\Vert Ax^{1}-b \bigr\Vert ^{2}+\frac {C_{3}}{2} \bigl\Vert x_{2}^{0}-x_{2}^{2} \bigr\Vert _{G_{0}}^{2}+\frac{C_{4}}{2}\beta \bigl\Vert A_{2}\bigl(x_{2}^{0}-x_{2}^{1} \bigr) \bigr\Vert ^{2}. $$

#### Proof

Setting $x=x^{*}$, $\lambda=\lambda^{*}$ in (37), and summing the resulted inequality over $k=1, 2, \ldots, t$, we have

42 $$\begin{aligned}& \sum_{k=1}^{t} \biggl[\theta \bigl(x^{k+1}\bigr)-\theta\bigl(x^{*}\bigr)+\bigl(x^{k+1}-x^{*} \bigr)^{\top}\bigl(-A^{\top}\lambda^{*}\bigr)+\frac{C_{2}}{2} \beta \bigl\Vert Ax^{k+1}-b \bigr\Vert ^{2} \biggr] \\& \quad \leq \frac{1}{2} \bigl\Vert v^{1}-v^{*} \bigr\Vert _{H}^{2}+\frac{C_{0}}{2}\beta \bigl\Vert Ax^{1}-b \bigr\Vert ^{2}+\frac{C_{3}}{2} \bigl\Vert x_{2}^{0}-x_{2}^{2} \bigr\Vert _{G_{0}}^{2}+\frac{C_{4}}{2}\beta \bigl\Vert A_{2}\bigl(x_{2}^{0}-x_{2}^{1} \bigr) \bigr\Vert ^{2}. \end{aligned}$$

Therefore, dividing (42) by *t* and using the convexity of $\theta(\cdot)$ lead to

43 $$ \theta(x_{t})-\theta\bigl(x^{*}\bigr)+ \bigl(x_{t}-x^{*}\bigr)^{\top}\bigl(-A^{\top}\lambda ^{*} \bigr)+\frac{C_{2}\beta}{2t}\sum_{k=1}^{t} \bigl\Vert A^{k+1}-b \bigr\Vert ^{2} \leq\frac{D}{t}, $$

where the constant *D* is defined by (41).

Compared (43) with (19), we only need to deal with the term $\frac{C_{2}\beta}{2t}\sum_{k=1}^{t}\|A^{k+1}-b\|^{2}$ on the left-hand side of (43). In fact, from the convexity of $\|\cdot\|^{2}$, we get

$$\begin{aligned}& \frac{C_{2}\beta}{2t}\sum_{k=1}^{t} \bigl\Vert A^{k+1}-b \bigr\Vert ^{2} \\& \quad =\frac{C_{2}\beta}{2}\sum_{k=1}^{t} \frac{1}{t} \bigl\Vert Ax^{k+1}-b \bigr\Vert ^{2} \\& \quad \geq\frac{C_{2}\beta}{2} \biggl\Vert A\frac{\sum_{k=1}^{t}x^{k+1}}{t}-b \biggr\Vert ^{2} \\& \quad =\frac{C_{2}\beta}{2} \Vert Ax_{t}-b \Vert ^{2}. \end{aligned}$$

Then, substituting the above inequality into (43), we get the desired result (40). This completes the proof. □

## Numerical results

We have established the convergence results of the IPS-ADMM in theory. In this section, by comparing the IPS-ADMM with the PS-ADMM [15], we are going to highlight its promising numerical behaviors in solving an image restoration problem: the total-variational denoising problem. All the codes were written by Matlab R2010a and all the numerical experiments were conducted on a THINKPAD notebook with Pentium(R) Dual-Core CPU@2.20 GHz and 4 GB RAM.

Below, we consider the total-variational (TV) denoising problem [29]:

44 $$ \min\frac{1}{2}\|y-b\|^{2}+\frac{\eta}{2}\| Dy\|_{1}, $$

where $D^{\top}=[D_{1}^{\top},D_{2}^{\top}]^{\top}$ is a discrete gradient operator with $D_{1}:\mathcal{R}^{n}\rightarrow\mathcal{R}^{n}$, $D_{2}:\mathcal {R}^{n}\rightarrow\mathcal{R}^{n}$ being the finite-difference operators in the horizontal and vertical directions, respectively; $\eta>0$ is the regularization parameter. Here, we set $\eta=5$.

Introducing an auxiliary variable $x\in\mathcal{R}^{2n}$, we can reformulate (44) as

45 $$ \begin{aligned} &\min\eta \|x\|_{1}+ \frac{1}{2} \|y-b\|^{2} \\ &\quad \mbox{s.t. } x-Dy=0, x\in\mathcal{R}^{2n}, y\in \mathcal{R}^{n}. \end{aligned} $$

Obviously, (45) is a special case of (1), and therefore the IPS-ADMM is applicable. Now, let us elaborate on how to derive the closed-form solutions for the subproblems resulted by the IPS-ADMM.

Set $P=\tau_{1}I_{2n}$, $G=\alpha\tau_{2}I_{n}-\beta D^{\top}D$. For given $(x^{k},y^{k},\lambda^{k})$, the first subproblem is

$${x}^{k+1}=\mathop{\operatorname{argmin}}_{x\in\mathcal{R}^{2n}} \biggl\{ \eta \Vert x \Vert _{1}-\bigl(\lambda^{k}\bigr)^{\top}\bigl(x-Dy^{k}\bigr)+\frac{\beta}{2} \bigl\Vert x-Dy^{k} \bigr\Vert ^{2}+\frac{1}{2} \bigl\Vert x-x^{k} \bigr\Vert _{P}^{2} \biggr\} , $$

which has a closed-form solution:

$${x}^{k+1}=\operatorname{shrink}_{1,2}\biggl( \frac{\tau_{1} x^{k}+\beta Dy^{k}+\lambda ^{k}}{\beta+\tau_{1}},\frac{\eta}{\beta+\tau_{1}}\biggr). $$

For given $x^{k+1}$, $y^{k}$, $\lambda^{k+\frac{1}{2}}$, the third subproblem is

$${y}^{k+1}=\mathop{\operatorname{argmin}}_{y\in\mathcal{R}^{n}} \biggl\{ \frac{1}{2} \Vert y-b \Vert ^{2}-\bigl(\lambda^{k+\frac{1}{2}} \bigr)^{\top}\bigl(x^{k+1}-Dy\bigr)+\frac{\beta}{2} \bigl\Vert x^{k+1}-Dy \bigr\Vert ^{2}+\frac{1}{2} \bigl\Vert y-y^{k} \bigr\Vert _{G}^{2} \biggr\} , $$

which has a closed-form solution:

$${y}^{k+1}=\frac{1}{1+\alpha\tau_{2}}\bigl(b-D^{\top}\lambda^{k+\frac {1}{2}}+\beta Dx^{k+1}+Gy^{k}\bigr). $$

For the IPS-ADMM, we set $\beta=1$, $\tau_{1}=0.001$, $\tau_{2}=1.01\beta\| D^{\top}D\|$, $\alpha=1.01c(r,s)$. For the PS-ADMM, we set $G=\tau_{2} I_{n}-\beta B^{\top}B$. The initialization is chosen as $x_{0}=0$, $y_{0}=b$, $\lambda_{0}=0$. The stopping criterion is the same as that in [2]:

$$\bigl\Vert x^{k+1}-Dy^{k+1} \bigr\Vert \leq \epsilon^{\mathrm{pri}} \quad \mbox{and}\quad \bigl\Vert \beta D \bigl(y^{k+1}-y^{k}\bigr) \bigr\Vert \leq \epsilon^{\mathrm{dual}}, $$

where $\epsilon^{\mathrm{pri}}=\sqrt{n}\epsilon^{\mathrm{abs}}+\epsilon ^{\mathrm{rel}}\max\{\|x^{k+1}\|,\|Dy^{k+1}\|\}$, and $\epsilon^{\mathrm{dual}}=\sqrt{n}\epsilon^{\mathrm{abs}}+\epsilon ^{\mathrm{rel}}\|y^{k+1}\|$ with $\epsilon^{\mathrm{abs}}=10^{-4}$ and $\epsilon^{\mathrm{rel}}=10^{-3}$. We use the following Matlab scripts to generate some synthetic data for (45) [21]:

$$\begin{aligned}& \mathtt{for\ j = 1:3} \\& \quad \mathtt{id = randsample(n,1)} ; \\& \quad \mathtt{id = randsample(n,1)} ; \\& \quad \mathtt{y(ceil(idx/2):idx) = k*y(ceil(idx/2):idx)} ; \\& \mathtt{end} \\& \quad \mathtt{b = y + randn(n,1)} ; \\& \quad \mathtt{e = ones(n,1)} ; \\& \quad \mathtt{D = spdiags([e -e], 0:1, n,n)} ; \end{aligned}$$

We list some numerical results in Table 1. Numerical results in Table 1 illustrate that the IPS-ADMM often performs much better than the PS-ADMM, though the difference between them only lies in the proximal parameter. Then, the numerical advantage of smaller proximal parameter is verified.

**Table 1:** **Comparison between the number of iterations (time in seconds) taken by PS-ADMM and IPS-ADMM for TV denoising problem**

***n***	**PS-ADMM** **(** ***r*** **,** ***s*** **)=(−0.3,1.2)**	**IPS-ADMM** **(** ***r*** **,** ***s*** **)=(−0.3,1.2)**	**Ratio (%)**	**PS-ADMM** **(** ***r*** **,** ***s*** **)=(0.3,1.2)**	**IPS-ADMM** **(** ***r*** **,** ***s*** **)=(0.3,1.2)**	**Ratio (%)**
100	176 (0.04)	94 (0.03)	0.53 (0.60)	149 (0.06)	97 (0.02)	0.65 (0.41)
200	213 (0.05)	107 (0.03)	0.50 (0.49)	180 (0.04)	117 (0.03)	0.65 (0.67)
300	189 (0.06)	104 (0.03)	0.55 (0.45)	160 (0.04)	105 (0.03)	0.66 (0.63)
400	47 (0.02)	24 (0.01)	0.51 (0.43)	40 (0.01)	27 (0.01)	0.68 (0.88)
500	99 (0.03)	54 (0.02)	0.55 (0.56)	84 (0.03)	56 (0.02)	0.67 (0.68)

## Conclusions

In this paper, a symmetric ADMM with indefinite proximal regularization for two-block linearly constrained convex programming is proposed. Under mild conditions, we have established the global convergence and the worst-case $\mathcal{O}(1/t)$ convergence rate in an ergodic sense of the new method. Some numerical results are given, which illustrate that the new method often performs better than its counterpart with positive definite proximal regularization. Note that this paper only discusses the symmetric ADMM with indefinite proximal regularization for the two-block separable convex problems. In the future, we shall study the ADMM-type method with indefinite proximal regularization for the multi-block case.
